# Specialists

**Published:** 1864-04

**Authors:** 


### EDITORIAL DEPARTMENT.

### SPECIALISTS.

We publish a communication under the head of correspondence, upon the subject of Epilepsy, and desire to call the attention of our readers, not only to what is said about the treatment of epilepsy, but to the general character of the communication. Newspaper puffs are offered editors of medical journals for re-publication upon the ground that the author, or *victim*' whichever you please, is not engaged in general practice, but pursues a specialty. Our correspondent we have no doubt is as professional, and meritorious as others, at least we have no reason to presume otherwise. His communication is not unlike others which have been received, and is published as an illustration of the expectations of specialists, who claim to be highly professional, belong to all the honorable medical societies and associations; in fellowship with the most honorable members of the profession, in every way above suspicion, so far as professional standing is concerned, and yet regard themselves at liberty to publish the most exaggerated statements as regards the cure of those under their care, and sometimes allow partial friends to make statements which would do credit to the most unblushing quackery. It is not uncommon for specialists, in good and regular standing in the profession, to make, or allow, newspaper puffs which are identical in character with those written and published by the most notorious charlatans and impostors. This may not be construed to say, that specialists are charlatans and impostors; for a physician may profitably cultivate one department of professional knowledge and thereby excel in that branch, but he is not on that account at liberty to disgrace himself and the profession by a resort to the very tricks, by which the pretender and the cheat, obtain temporary fame.

Specialties may be all very well, while men pursue them in a proper manner, but temptation has so often overcome those who have thus engaged, that we regard the grace of God as hardly sufficient to save any, howbeit some may possibly escape; not more than enough however to prove the general rule.

With clamor and pretesnion, the very temples of medical knowledge are defamed; they "offer their unholy sacrifices upon consecrated altars." Local medical societies, national associations and medical journals are bombarded, and partially taken, while the profession are informed, that, if engaged in general practice, they would not advertise; meanwhile the very reason they are not in general practice, is because they have resolved to "gain the rewards of diligence without suffering its fatigues"—to make appeal to the people in expectation that their superior attainments will be the sooner appreciated.

We do not oppose the pursuit of any one division or branch of medical practice. It is unquestionably proper to give attention exclusively to any department most preferred, and we have no doubt that those who actually do study, and treat exclusively one class of disease, often become better acquainted with its character, and more competent in treatment than the general practitioner; but that large class who make a specialty of treating every known disease, when presented, and of paying special attention to them all, or who advertise one specialty in particular, and all others in general, cannot reasonably be supposed to know more than others, or treat more successfully any of the diseases concerning which they would have us believe them so eminently intelligent. It is not alone, this open and baseless pretension we dislike, but we are often shocked at the claims of men, who, like our correspondent, very likely possess, superior knowledge, and wholly and honestly pursue some one branch of medical knowledge. There are many things to be said in favor of such men; many truths have been discovered by them; much has medical knowledge been advanced by such concentrated effort. Because they have merit, we shudder that it should be made known by the same processes by which those who have none, acquire fame. We cannot endure, that imposture and merit wear the same dress, pass by the same name, and keep the same company.

We desire in this connection to call attention to the resolutions upon this subject, presented to, and adopted by the State Medical Society, a copy of which will be seen in the brief synopsis of its proceedings in our last Journal. These resolutions were presented by the most intelligent, judicious, experienced, and impartial physicians in the State, and were unanimously adopted by the Society. We most cordially approve the sentiments expressed, and hope that those who would be respected by the profession will have sufficient regard for professional honor to conform to the letter and spirit of these resolutions.

